# The Strong *In Vivo* Anti-Tumor Effect of the UIC2 Monoclonal Antibody Is the Combined Result of Pgp Inhibition and Antibody Dependent Cell-Mediated Cytotoxicity

**DOI:** 10.1371/journal.pone.0107875

**Published:** 2014-09-19

**Authors:** Gábor Szalóki, Zoárd T. Krasznai, Ágnes Tóth, Laura Vízkeleti, Attila G. Szöllősi, György Trencsényi, Imre Lajtos, István Juhász, Zoltán Krasznai, Teréz Márián, Margit Balázs, Gábor Szabó, Katalin Goda

**Affiliations:** 1 Department of Biophysics and Cell Biology, Faculty of Medicine, University of Debrecen, Debrecen, Hungary; 2 Department of Obstetrics and Gynecology, Faculty of Medicine, University of Debrecen, Debrecen, Hungary; 3 Department of Preventive Medicine, Faculty of Medicine, University of Debrecen, Debrecen, Hungary; 4 Department of Physiology, Faculty of Medicine, University of Debrecen, Debrecen, Hungary; 5 Department of Nuclear Medicine, Faculty of Medicine, University of Debrecen, Debrecen, Hungary; 6 Department of Dermatology, Faculty of Medicine, University of Debrecen, Debrecen, Hungary; 7 Department of Surgery and Operative Techniques, Faculty of Medicine, University of Debrecen, Debrecen, Hungary; Hungarian Academy of Sciences, Hungary

## Abstract

P-glycoprotein (Pgp) extrudes a large variety of chemotherapeutic drugs from the cells, causing multidrug resistance (MDR). The UIC2 monoclonal antibody recognizes human Pgp and inhibits its drug transport activity. However, this inhibition is partial, since UIC2 binds only to 10–40% of cell surface Pgps, while the rest becomes accessible to this antibody only in the presence of certain substrates or modulators (e.g. cyclosporine A (CsA)). The combined addition of UIC2 and 10 times lower concentrations of CsA than what is necessary for Pgp inhibition when the modulator is applied alone, decreased the EC_50_ of doxorubicin (DOX) in KB-V1 (Pgp^+^) cells *in vitro* almost to the level of KB-3-1 (Pgp^-^) cells. At the same time, UIC2 alone did not affect the EC_50_ value of DOX significantly. In xenotransplanted severe combined immunodeficient (SCID) mice co-treated with DOX, UIC2 and CsA, the average weight of Pgp^+^ tumors was only ∼10% of the untreated control and in 52% of these animals we could not detect tumors at all, while DOX treatment alone did not decrease the weight of Pgp^+^ tumors. These data were confirmed by visualizing the tumors *in vivo* by positron emission tomography (PET) based on their increased ^18^FDG accumulation. Unexpectedly, UIC2+DOX treatment also decreased the size of tumors compared to the DOX only treated animals, as opposed to the results of our *in vitro* cytotoxicity assays, suggesting that immunological factors are also involved in the antitumor effect of *in vivo* UIC2 treatment. Since UIC2 binding itself did not affect the viability of Pgp expressing cells, but it triggered *in vitro* cell killing by peripheral blood mononuclear cells (PBMCs), it is concluded that the impressive *in vivo* anti-tumor effect of the DOX-UIC2-CsA treatment is the combined result of Pgp inhibition and antibody dependent cell-mediated cytotoxicity (ADCC).

## Introduction

One of the most common causes of cancer chemotherapy failure is the development of resistance against chemotherapeutic agents. In most cases the tumor cells are either intrinsically resistant, or become resistant in the course of chemotherapy, to a broad spectrum of chemotherapeutic agents, including compounds they have never met before [1]. This phenomenon is called multidrug resistance (MDR) and it is often associated with high-level expression of active transporter proteins belonging to the ATP Binding Cassette (ABC) super-family, such as ABCB1 (MDR1, P-glycoprotein, Pgp), ABCC1 (MRP1, multidrug resistance protein 1) or ABCG2 (BCRP, breast cancer resistance protein)[2], [3]. Pgp was the first transporter described in connection with multidrug resistance, and it seems to have the most significant role in clinical cases [3].

The Pgp molecule consists of two almost identical halves connected by a 75 amino acid long intracellular linker region. Both halves comprise six membrane spanning α-helices forming a transmembrane domain (TMD) and a nucleotide binding domain (NBD). The two TMDs define the substrate binding sites and the translocation pathway, allowing the protein to transport various hydrophobic compounds out of the cells [4]. The overall energy requirement of drug efflux is covered by ATP hydrolysis conducted by the two NBDs (for possible models, see e.g. Senior [5], Ambudkar et al. [6]).

Pgp is generally expressed in tissues having barrier functions (e.g., in endothelial cells of the blood-brain barrier, in hepatocytes, in epithelial cells of the kidney and the intestines) and it is suggested to have an important role in protection of the body from toxic substances [2], [3], [7]). However, the loss of the *abcb1a/b* genes in mice (homologues of the human *ABCB1* gene) is not accompanied by major physiological consequences [8], [9]; hence, inhibition of Pgp molecules may be a plausible strategy of overcoming drug resistance without serious side effects. The classical pharmacological approach involves co-administration of the cytotoxic compounds that are substrates of Pgp with pump inhibitors, to increase the accumulation of the former into the tumor cells. Unfortunately, Pgp inhibitors often induce unpredictable and intolerable pharmacokinetic interactions and toxicity through inhibiting other drug transporters or cytochrome P450, by changing the clearance and metabolism of the co-administered chemotherapeutic agents [10]–[12]

Several monoclonal antibodies (mAb) recognizing extracellular epitopes have been developed against Pgp. A few of them (e.g., MRK16, MRK17, MC57, HYB-241, and UIC2) are thought to recognize discontinuous conformation sensitive epitopes. Upon binding, these antibodies can partially inhibit Pgp mediated drug transport *in vivo* and *in vitro* [13]–[16]. However, this inhibitory effect is often weak [13]–[18], its extent may depend on the transported substrate [15], [17], [18], and it is variable even in the case of the same substrate according to general experience.

UIC2 is an IgG2a isotype mouse monoclonal antibody raised against human Pgp. It recognizes a complex epitope involving at least the first [19] and the third extracellular loops of the protein [20]. In the absence of Pgp substrates and modulators UIC2 can bind only to 10–40% of cell surface Pgps, while the rest adopts the UIC2 binding conformation only in the presence of a distinct group of substrates or modulators (e.g., cyclosporine A (CsA), SDZ PSC 833, vinblastine and paclitaxel [21]–[23]. In previous studies we have demonstrated that the UIC2 antibody itself completely inhibits Pgp function when it is applied together with any of the above Pgp modulators added at low, sub-inhibitory concentrations [22]. The above phenomenon was also confirmed *in vitro*, by measuring the cellular accumulation of various fluorescent Pgp substrates including DNR, R123, calcein and a radioactive tracer ^99m^Tc-Mibi [22]. In line with the above, combined treatment with either CSA + UIC2 or paclitaxel + UIC2 specifically decreased the rate of glucose metabolism in Pgp^+^ cells, as measured in 2-[^18^F]fluoro-2-deoxy-D-glucose (^18^FDG) accumulation experiments, also suggesting that Pgp inhibition with concomitant decrease of energy consumption has occurred [23]. On the other hand, we also demonstrated in xenotransplanted severe combined immunodeficient (SCID) mice that UIC2 could readily penetrate into the compact solid tumors, intensively staining cell surface Pgps and increasing daunorubicin accumulation in the Pgp^+^ tumors to the level of the Pgp^-^ ones [22].

In the present study we tested whether the combined treatment with CsA and UIC2 mAb can potentiate the anti-tumor effect of doxorubicin (DOX) in Pgp^+^ tumors to achieve clinically relevant reduction in tumor size, applying the above experimental model [22]. Tumor growth was followed by weighing the mass of the tumors in sacrificed animals and also *in vivo* on the basis of ^18^FDG accumulation. In the latter case a small-animal Positron Emission Tomography (PET) camera was applied to visualize tumors on the basis of their increased rate of glucose metabolism [24]–[26]. Our data demonstrate that the combined application of a class of modulators (including CsA) used at sub-inhibitory concentrations and of the UIC2 antibody may serve as an effective tool for blocking the growth of Pgp expressing tumors.

## Materials and Methods

### Ethics Statement

The experiments using human blood were done with the approval of the Scientific and Research Ethics Committee of the Medical Research Council (ETT TUKEB, permission number: 25364-1/2012/EKU (449/P1/12.)). Written informed consent was obtained from donors prior to blood donation, and their data were processed and stored according to the principles expressed in the Declaration of Helsinki.

In animal experiments the *Principles of Laboratory Animal Care* (National Institute of Health) was strictly followed, and the experimental protocol was approved by the Laboratory Animal Care and Use Committee of the University of Debrecen (Permission Numbers: 26/2006/DE-MAB and 122/2009/DE-MAB).

### Cell Lines

KB-3-1 human epidermoid carcinoma cell line and KB-V1, its Pgp positive counterpart were used in the experiments (obtained from Michael Gottesman's lab, NIH, Bethesda) [27], [28]. The cells were grown as monolayer cultures at 37°C in Dulbecco's modified Eagle's medium (DMEM) containing 4.5 g/l glucose and supplemented with 10% heat-inactivated fetal bovine serum (FBS), 2 mM L-glutamine and 25 µM/ml gentamycin. The KB-V1 cells were cultured in the presence of 180 nM vinblastine until 3 days before their use. The viability of the cells in our experiments was always higher than 90%, as assessed by the trypan blue exclusion test. The cells were regularly checked for mycoplasma by the Plasmo Test mycoplasma detection kit (San Diego, CA) and found to be negative.

### Chemicals

All the Pgp substrates, modulators, cell culture media and supplements were from Sigma–Aldrich (Budapest, Hungary). The UIC2, 15D3, 5D3 and QCRL-3 mAbs were purified from the supernatants of hybridoma cell lines using affinity chromatography. The hybridoma cell lines were purchased from the American Type Culture Collections, Manassas, VA, USA), except the 5D3 hybridoma cell line, which was a kind gift from Brain P. Sorrentino (Division of Experimental Hematology, Department of Hematology/Oncology, St. Jude Children's Research Hospital, Memphis, Tennessee). The mAb preparations were>97% pure by SDS/PAGE. The glucose analogue 2-[^18^F]fluoro-2-deoxy-D-glucose (^18^FDG) was synthesized and labeled with the positron-decaying isotope ^18^F according to Hamacher et al. [29].

### Indirect immunofluorescence

For detection of Pgp and ABCG2 living cells (10^6^ cells/ml) were incubated in the presence of 30 µg/ml 15D3 anti- Pgp mAb or 2 µg/ml 5D3 anti-ABCG2 mAb for 30 min at 37°C. For measurement of MRP1 (ABCC1) expression cells were fixed and permeabilised with 1% para-formaldehyde and 0.1% TritonX-100 in PBS (15 min; 4°C) and then labeled with 2 µg/ml QCRL-3 mAb (30 min; 4°C). After two washes with ice-cold PBS containing 1% bovine serum albumin (BSA-PBS), cells were incubated with goat anti-mouse IgG (0.5 µg/ml CruzFluor 647 (CFL647-GaMIgG), Santa Cruz Biotechnology, Inc., Texas) for 30 min at 4°C. Fluorescence intensities were detected using a Becton Dickinson FACSAria III Cell Sorter (Becton Dickinson) measuring the 633 nm/660±20 nm fluorescence intensities. Fluorescence signals were collected in logarithmic mode and the cytofluorimetric data were analyzed by the BDIS CELLQUEST (Becton Dickinson) software.

To detect Pgp expression in tumors 5-µm-thick cryosections were prepared, dried at room temperature and fixed in pre-cooled acetone (-20°C) for 10 min. Sections were then washed with PBS and blocked with 1% BSA-PBS for 20 minutes to avoid non-specific labeling and further incubated at room temperature with the UIC2 (10 µg/ml) mouse mAb for 60 min. To visualize the binding of the primary antibody an Alexa-488 conjugated goat anti-mouse IgG (A488-GaMIgG, Invitrogen) was used at 1∶1000 dilution. Negative controls were obtained by omitting the primary antibody.

A Zeiss LSM 510 confocal laser-scanning microscope was used for the measurements. Alexa-488 was excited at the 488 nm line of an argon-ion laser. Fluorescence was detected through a 505–550 nm band pass filter. Images of 512×512 pixels were obtained in extended focus mode, through a 63× (numerical aperture = 1.4) Plan-Apochromat oil immersion objective.

### *In vitro* cytotoxicity tests

Cells were seeded in 96-well plates at a cell density of 5×10^3^ cells/well. 24 hours later DOX was added at different concentrations with CsA and/or UIC2 mAb or the modulator alone, and the plates were further incubated for 72 h at 37°C. The cell viability was determined using the AlamarBlue assay (Serotec, UK) measuring the 530/590 nm fluorescence intensity of the dye in an automated microplate reader (BioTec Synergy HT, US). The fluorescence intensities of the samples were normalized to the fluorescence of the untreated (DOX, antibody and CsA free) control sample, and plotted as a function of DOX concentration. Dose-response curves were fitted, and EC_50_ values were calculated by SigmaPlot 12.0 programme (Systat Software, Inc., USA).

### *In vitro* antibody-dependent cell-mediated cytotoxicity (ADCC) assay

ADCC experiments were performed as described previously [30] with minor modifications. Human peripheral blood mononuclear cells (PBMCs) were prepared from the blood of healthy donors by Ficoll (Histopaque-1077, Sigma-Aldrich, Budapest) density gradient centrifugation. The PBMC rich fraction (effector cells) was washed three times and re-suspended in DMEM containing 10% FCS. After trypsinization, KB-V1 and KB-3-1 (target cells) cells were labeled with 5(6)-carboxyfluorescein diacetate N-succinimidyl ester (CFDA-SE) at a concentration of 10 µM at 37°C for 10 min. Then, the cells were washed thrice with DMEM containing 10% FCS and 1% BSA to remove unbound CFDA-SE and finally re-suspended in DMEM containing 10% FCS. 1.5×10^5^ target cells were mixed with effector cells at target/effector cell ratios of 1∶5, 1∶10, 1∶50 and 1∶100 in a final volume of 1 ml. Samples were incubated in the absence or presence of 0.1 µM CsA and/or 20 µg/ml UIC2 at 37°C for 8 hours in CO_2_ incubator. After incubation the cells were washed and then re-suspended in ice cold phosphate-buffered saline (PBS), containing 8 mM glucose and 5 µg/ml propidium iodide (PI) and were analyzed using a Becton Dickinson FACScan flow cytometer (Mountain View, CA). The labeling distinguishes four populations of cells as it is demonstrated in Fig. S1: **1**. living target cells in green (CFDA-SE positive cells); **2**. dead target cells in green and red (CFDA-SE and PI double positive cells); **3**. dead effector cells in red (PI positive cells), and **4**. live effector cells, which remain unstained. The negative control sample did not contain PBMCs, while tumor cells killed by 4% para-formaldehyde served as the positive control. The percentage of killed target cells was calculated dividing the number of PI and CFDA-SE double positive cells (dead cells) by the number of the CFDA-SE positive cells.

### *In vitro* complement-dependent cytotoxicity (CDC) assay

Samples containing 2.5×10^5^ KB-V1 or KB-3-1 cells and 20 µg/ml UIC2 mAb in the presence or absence of 0.1 µM CsA were treated with freshly prepared human serum at different dilutions in DMEM medium. The samples were incubated for 4 hours in CO_2_ incubator at 37°C and then stained with 5 µg/ml PI. The percentage of the PI positive dead cell was determined by a Becton Dickinson FACScan flow cytometer (Mountain View, CA).

The hemolytic complement activity of the human serum samples was determined applying sheep red blood cells sensitized with a rabbit stroma antibody (SSRBC) kindly provided by Attila Bácsi (Inst. of Immunology, University of Debrecen, Faculty of Medicine). 50 µl of 1% SSRBC was mixed with different dilutions of human serum or heat inactivated human serum (inactivated at 56°C for 30 min) and incubated for 30 min at 37°C. After centrifugation the absorbance of the supernatants was measured at 541 nm by BioTek Synergy HT plate reader. The HC_50_ value where 50% of the RBCs were lysed was determined for the human sera and found to be normal.

### Animal model and study design

Adult (10 to 12 week-old), pathogen-free B-17 severe combined immunodeficiency (SCID) mice were used in this study [31]. Animals were housed under pathogen free circumstances at a temperature of 26±2°C, with 50±10% humidity and artificial lighting with a circadian cycle of 12 h. The food and drinking water (sterilized by autoclaving) were available ad-libitum to all animals.

SCID mice were injected subcutaneously with KB-3-1 (Pgp^-^; 1.5×10^6^ cells in 150 µl sterile phosphate-buffered saline (PBS)) on the left and KB-V1 (Pgp^+^; 3×10^6^ cells in 150 µl sterile PBS) cells on the right thighs. To obtain approximately similar tumor sizes in case of KB-V1 and KB-3-1 cells we had to inject double number of KB-V1 cells, because of their slower cell proliferation rate. In another experiment we grafted four tumors per animal in order to limit the number of animals and to maximize the number of tumors imaged. In this case each animal received two injections in the shoulders and two in the upper part of the thighs. Tumors were grown for 4 days and then the mice were treated with DOX alone (5 mg/kg, i.v.), or DOX combined with either UIC2 mAb (5 mg/kg, i.v.) or CsA (Sandimmun, Novartis, Basel, Switzerland; 10 mg/kg i.p.) or both. The animals were killed 8 days after treatment with chemotherapy by cervical dislocation and the tumors were removed to weigh them and then they were snap frozen in liquid nitrogen and stored at -70°C till mRNA expression analysis.

### mRNA expression analysis

The frozen tumor sections were equilibrated in 10 volumes pre-chilled RNAlater-ICE solution (Applied Biosystem, CA) at -20°C overnight to protect RNA from degradation and then total RNA was isolated using the RNeasy Mini kit (Qiagen Inc., CA) according to the protocol. RNA quantity was determined by NanoDrop ND-1000 UV-Vis Spectrophotometer (NanoDrop Technologies, Wilmington, DE). RNA was then subjected to reverse transcription-real time quantitative polymerase chain reaction (RT-qPCR) using the Taqman assay with stocked primers and probes (Applied Biosystem, CA). Pgp mRNA expression was normalized to the human glyceraldehyde-3-phosphate dehydrogenase (GAPDH) expression.

### Small-animal PET imaging using ^18^FDG

After the implantation of tumor cells ^18^FDG PET scans were repeated at different time points. Prior to PET measurements, mice were fasted overnight. On the day of PET imaging mice were pre-warmed to a body temperature of 37°C and this temperature was maintained throughout the uptake and scanning period to minimize the visualization of brown fat. Mice were injected via the tail vein with 5.5±0.2 MBq of ^18^FDG. 40 min after tracer injection animal were anaesthetized by 3% isoflurane with a dedicated small animal anesthesia device and then 20 min long static single-frame PET scans were acquired using a small-animal PET scanner (MiniPET-II, Department of Nuclear Medicine, Faculty of Medicine, University of Debrecen) to visualize the tumors.

The MiniPET-II system is a dedicated small animal PET scanner developed with the help of a Hungarian project. The MiniPET-II scanner consists of 12 detector modules including LYSO (Cerium Doped Lutetium Yttrium Orthosilicate) scintillation crystal blocks and position sensitive photo multiplier tubes. Each crystal block comprises 35×35 pins of 1.27×1.27×12 mm size. Scanner normalization and random correction were applied on the data and the images were reconstructed with the standard maximum likelihood expectation maximization iterative algorithm. The pixel size was 0.27×0.27×1.35 mm and the spatial resolution varies between 1.4 to 2.1 mm from central to 25 mm radial distances. The system sensitivity is 11.4%.

### ^18^FDG-PET data analysis

The ^18^FDG uptake was expressed in terms of standardized uptake values (SUVs) and tumor to muscle (T/M) ratios. Ellipsoidal 3-dimensional regions of interest (ROI) were manually drawn around the edges of the tumor xenografts by visual inspection using BrainCad software (Institute of Nuclear Medicine, University of Debrecen). The standardized uptake value (SUV) was calculated as follows: SUV = [ROI activity (Bq/ml)]/[injected activity (Bq)/animal weight (g)], assuming a density of 1 g/cm^3^. The T/M ratios were computed as the ratio of the mean activity in the tumor volume of interest (VOI) and the background (muscle) VOI.

### Statistical analysis

The displayed data are the means ± SD of the results of at least three independent experiments. Data have been analyzed using SigmaPlot 12.0 programme (Systat Software, Inc., USA) and IBM SPSS Statistics 20 (IBM Corp., USA). Statistical significance was assessed using analysis of variance (ANOVA), applying Holm-Sidak method for post hoc pair-wise comparison of the different samples. In the case of unequal variances Dunnett T3 post hoc pair-wise comparison method was used. Differences were considered to be significant at P<0.05.

## Results

### *In vitro* cytotoxicity measurements

**Figure 1:**
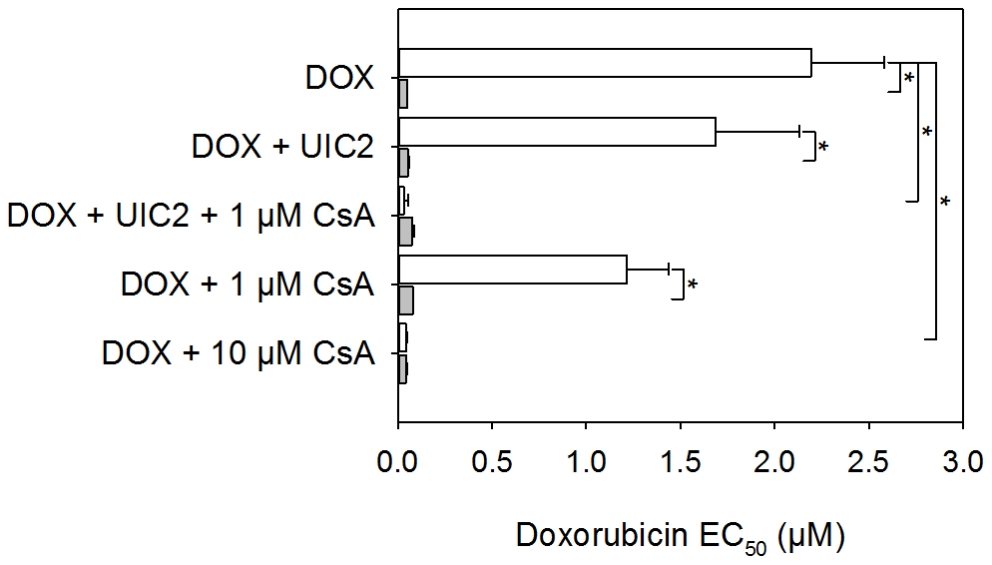
Cytotoxic effect of DOX in KB-3-1 (Pgp^-^; grey bars) and KB-V1 (Pgp^+^; empty bars) cells and its potentiation by treatments with CsA (used at the indicated concentrations) and/or UIC2 mAb (20 µg/ml). EC_50_ values were calculated by fitting the dose-response curves. Values are means (± SD) of 3 independent experiments. Statistically significant differences are shown by * (P<0.05).

KB-V1 cells express Pgp at high level, while other drug transporting ABC proteins ABCG2 and ABCC1 were not detectable by means of indirect immunofluorescence and flow cytometry (Fig. S2). KB-3-1 cells do not express any of the above ABC transporters at measurable level (Fig. S2). In accordance with the high Pgp expression level of KB-V1 cells the EC_50_ value of DOX was 2.19±0.39 µM in these cells, while it was only 44±3 nM in KB-3-1 (Pgp^-^) cells (Fig. 1). In KB-V1 cells CsA co-treatment decreased the EC_50_ value of DOX in a dose dependent manner, while UIC2 had only a weak, statistically not significant effect. Interestingly, the combined treatment of KB-V1 cells with 1 µM CsA and a saturating concentration of UIC2 mAb decreased the EC_50_ value to 33±19.7 nM, which could be achieved by 10 times higher CsA concentration when it was applied alone (Fig. 1). In contrast, administration of the UIC2 mAb and 1 µM CsA to cultures of KB-3-1 cells, simultaneously or alone, had no significant effects on their DOX sensitivity.

### mRNA expression analysis of Pgp in the tumor xenografts and cells used for grafting the tumors

**Figure 2:**
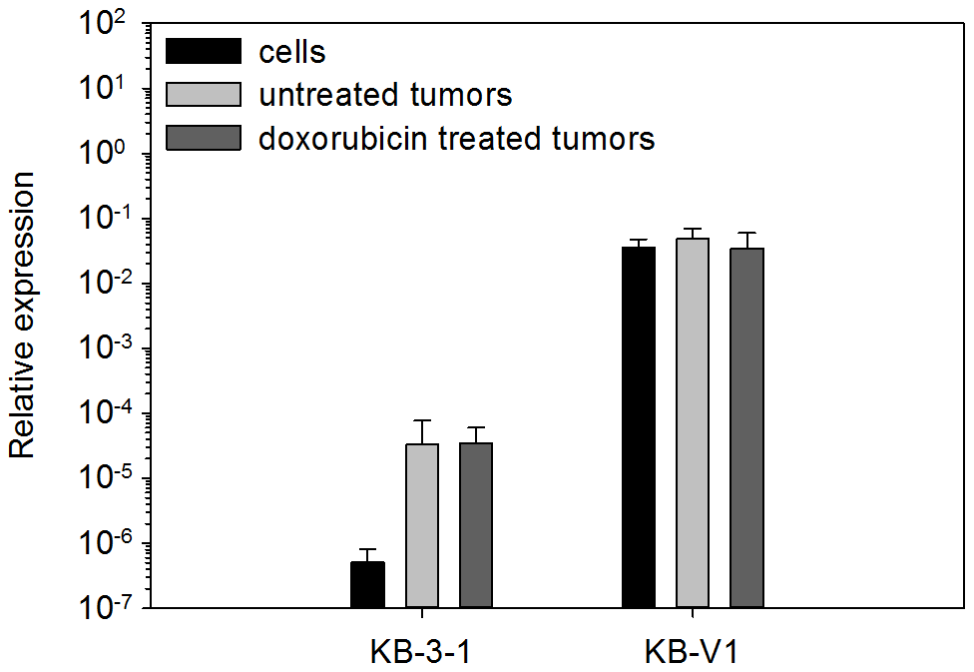
Relative Pgp mRNA expression levels of the KB-V1 and KB-3-1 tumors and the cells applied for grafting the tumors. Pgp expression levels are normalized to the expression level of GAPDH mRNA (mean ± SD, n = 5). The Pgp expression levels of inoculated KB-V1 cells and tumors were significantly different from those of the KB-3-1 cells and tumors (P<0.01), while the Pgp expression levels of tumors were not statistically different from those of the cells they originated from.

Based on the above *in vitro* results, we have designed *in vivo* experiments to test the effectiveness of the combined treatment with low dose of CsA, UIC2 and DOX. SCID mice were inoculated with KB-V1 and KB-3-1 cells, respectively. Palpable subcutaneous tumors developed in 10–12 days. Their Pgp expression was compared to that of the tumor cells used for grafting the tumors. Since the immunofluorescence intensities in frozen tissue sections and cell monolayers are not directly comparable, Pgp expression was examined at the mRNA level. In the KB-3-1 tumors a well detectable ∼60-fold increment of Pgp mRNA levels occurred compared to the inoculated cells, while the Pgp mRNA level of the KB-V1 tumors did not change upon proliferation of the inoculated cells (Fig. 2). The Pgp mRNA levels proved to be at least three orders of magnitude higher in the KB-V1 tumors compared to the KB-3-1 xenografts (see Fig. 2). Thus, the KB-V1 tumor xenografts retained their MDR phenotype as it was also proved by indirect immunofluorescent labeling (see Fig. S3), while the KB-3-1 cells continued to express Pgp at very low levels (not detectable by immunofluorescence, Fig. S3) in the developed tumors on the time scale of the *in vivo* experiments.

### ^18^FDG accumulation in xenotransplanted tumors

**Figure 3:**
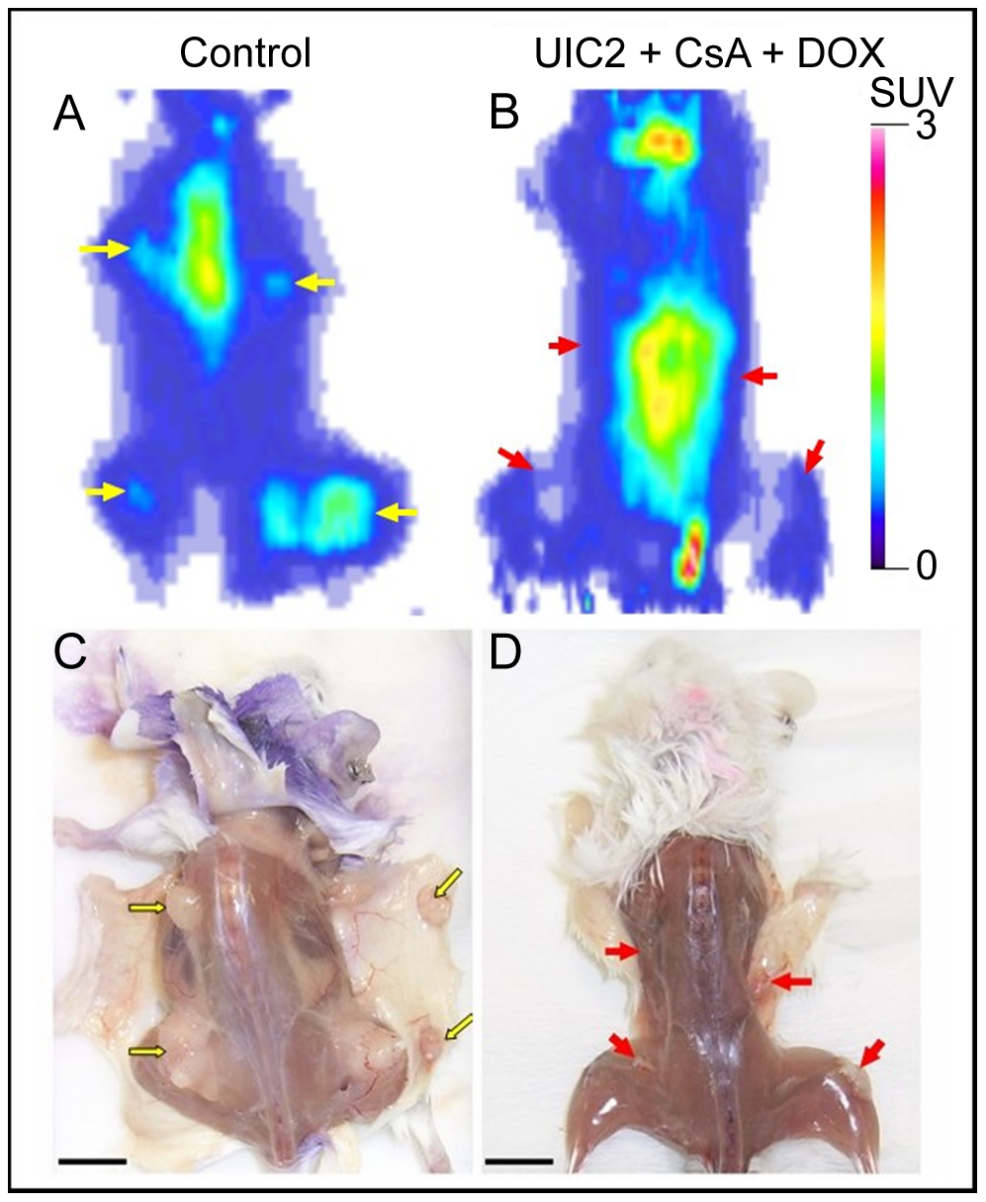
Effect of combined treatment with DOX+CsA+UIC2 on the KB-V1 and KB-3-1 tumors visualized *in vivo* by ^18^FDG-miniPET. Coronal section of ^18^FDG-miniPET image of a representative control (*A*) and a DOX-CsA-UIC2-treated tumor bearing mouse (*B*). Standardized uptake values (SUV) are calculated as described in Materials and Methods. The sites of tumor cell inoculation are shown by arrows (KB-V1 tumors, right side arrows; KB-3-1 tumors, left side arrows). Below: autopsies of the same control (*C*) and treated (*D*) animals. Bar: 10 mm.

Grafted tumors were grown for 4 days, then the mice were treated with DOX alone (5 mg/kg, i.v.) or DOX combined with either the UIC2 mAb (5 mg/kg, i.v.) or CsA (10 mg/kg, i.p.), or both. For an *in vivo* visualization of their effects, miniPET-^18^FDG accumulation measurements were performed, carrying out the scans 6–8 days after the above treatments. Fig. 3 shows representative ^18^FDG accumulation scans and the anatomical pictures of untreated (Fig. 3*A and C*) and DOX-UIC2-CsA treated (Fig. 3*B and D*) SCID mice bearing Pgp^+^ (KB-V1, right shoulder and thigh) and Pgp^-^ (KB-3-1, left shoulder and thigh) tumor xenografts. At the time of the measurements the tumors of untreated mice were 5–8 mm in diameter (6.43±1.13 mm mean ± SD, KB-3-1; 6.0±1.15 mm mean ± SD, KB-V1), and well-detectable on the basis of their increased rate of glucose metabolism.

The tumor (T) to skeletal muscle (M) ^18^FDG accumulation ratio (T/M) was 4.2±0.6 in the case of Pgp^-^ and 4.8±0.7 for the Pgp^+^ tissues (n = 7, ± SD), indicating significantly higher rate of glucose consumption in tumors compared to the muscle cells in both cases.

No visible or palpable tumors developed in 20% of the animals treated with the combination of DOX-UIC2-CsA. This observation was confirmed by the mini-PET scans, since no significant ^18^FDG accumulation was observed at the sites of tumor cell inoculation in these animals, as reflected by the T/M ^18^FDG accumulation ratios being close to 1 (T/M = 1.1±0.2 for Pgp^+^ and T/M = 0.97±0.1 for Pgp^-^ tumors; (n = 4)).

### Effect of DOX treatment combined with UIC2 and/or a low dose of CsA on the weights of grafted tumors

**Figure 4:**
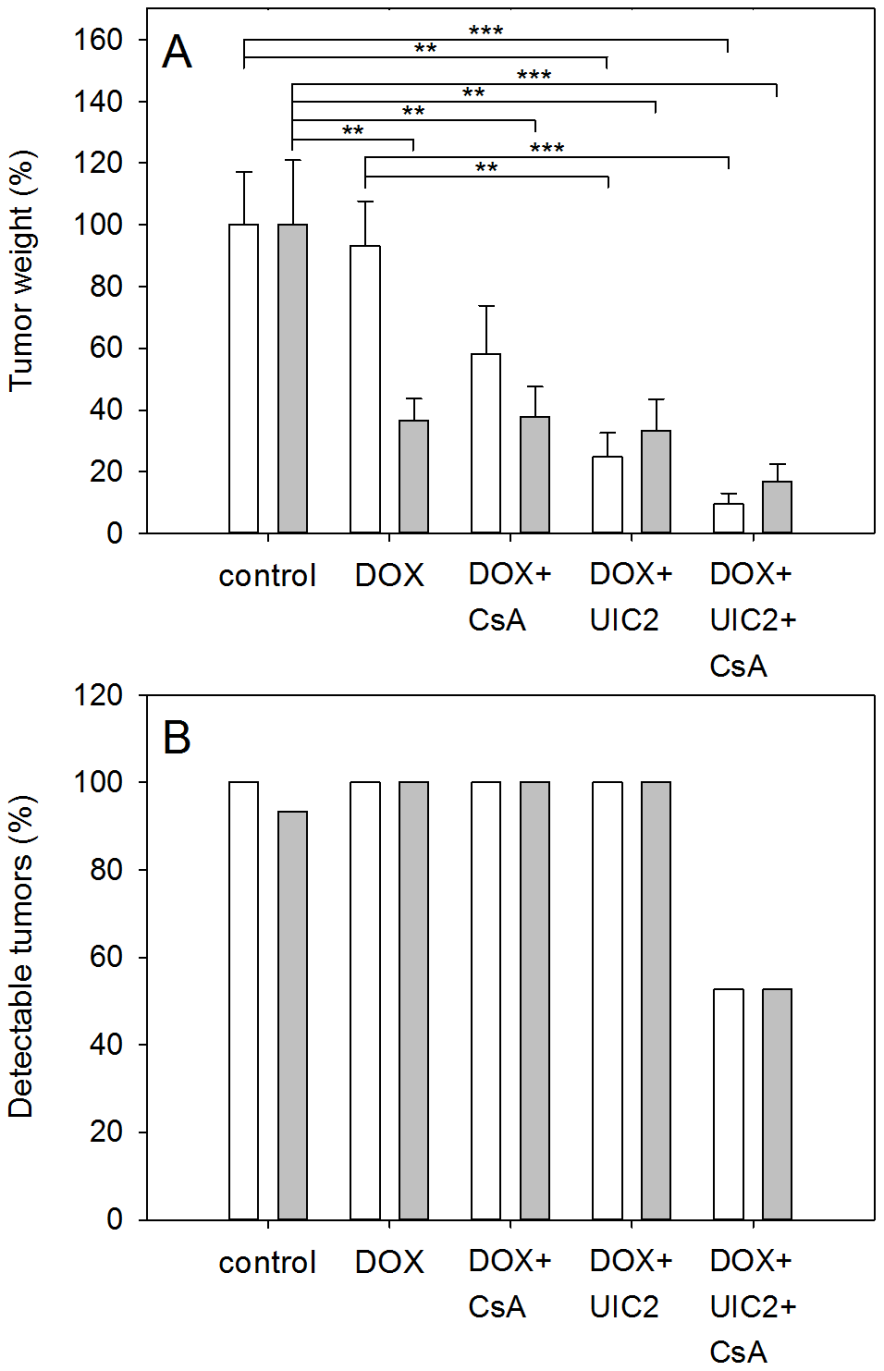
Effect of treatment with DOX combined with UIC2 and/or a low dose of CsA on the weights of the grafted tumors (*A*) and on the percentage of the detectable tumors in the different treatment groups (*B*). Tumor weights were expressed as a percentage of the average weight of the tumors of untreated animals measured at the time of the termination of the experiment (mean values ± SEM, n = 8). Statistically significant differences relative to the untreated control and the DOX-only treated groups are marked with *: P<0.05; **: P<0.01; ***: P<0.001). Grey bars: KB-3-1 (Pgp^-^) and empty bars: KB-V1 (Pgp^+^) tumors.

**Figure 5:**
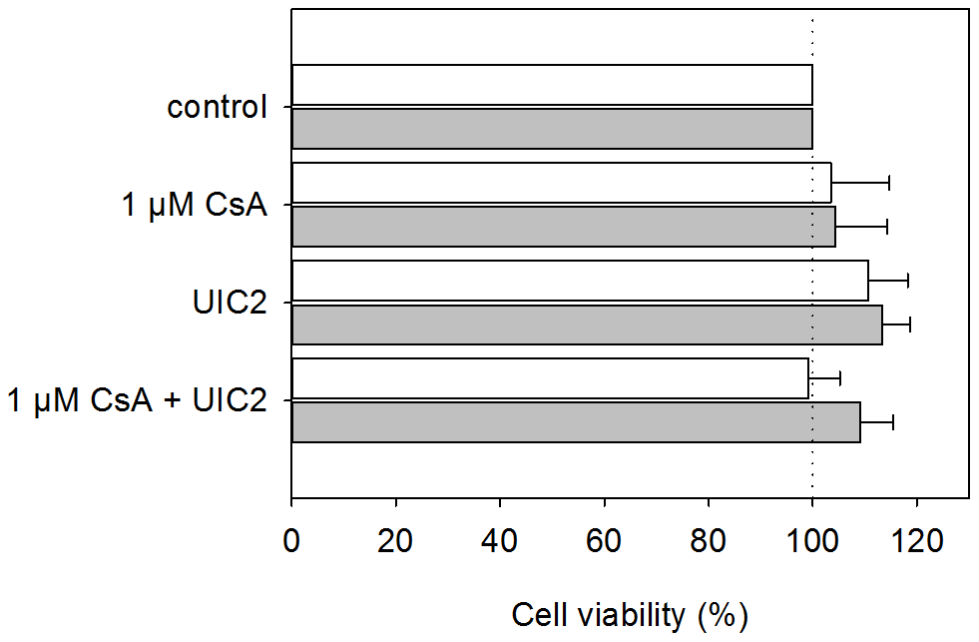
Effect of the UIC2 mAb treatment on the *in vitro* viability of KB-3-1 (Pgp^-^; grey bars) and KB-V1 cells (Pgp^+^; empty bars) in the presence or absence of 1 µM CsA. Cell viability was expressed as percentage of the untreated control. Values are means (± SD) of three independent experiments.

Eight days after chemotherapy, the animals were sacrificed by cervical dislocation and the tumors were removed to weigh them. As it is shown in Fig. 4*A*, DOX treatment alone was almost ineffective in the case of KB-V1 tumors, while the weight of the KB-3-1 tumors decreased considerably. Co-administration of 10 mg/kg CsA decreased the size of the KB-V1 tumors only mildly and did not affect the KB-3-1 tumors. Combined treatment with DOX-CsA-UIC2 decreased the mean weight of the KB-V1 tumors 9 fold compared to the animals treated with DOX alone. The combined treatment also decreased the mean weight of the KB-3-1 tumors, but not in a statistically significant extent. Importantly, only 52% of the grafted Pgp^+^ or Pgp^-^ tumors developed into detectable tumors in the DOX-CSA-UIC2 treated animals (see Fig. 4*B*), and 20% of the animals remained completely tumor-free. In contrast, we always detected tumors in the other treatment groups. Co-administration of UIC2 and DOX also decreased tumor size significantly compared to DOX alone in the case of KB-V1 tumors. This finding, in view of the fact that UIC2 treatment alone does not affect the EC_50_ value of DOX in *in vitro* cytotoxicity tests (Fig. 1), suggested to us that the growth inhibitory effect of the antibody is not exclusively due to Pgp inhibition. UIC2 binding did not affect the *in vitro* cell viability significantly (Fig. 5); therefore, the contribution of the immune system, that is partly functional in the SCID mice, was tested.

### *In vitro* ADCC and CDC measurements

**Figure 6:**
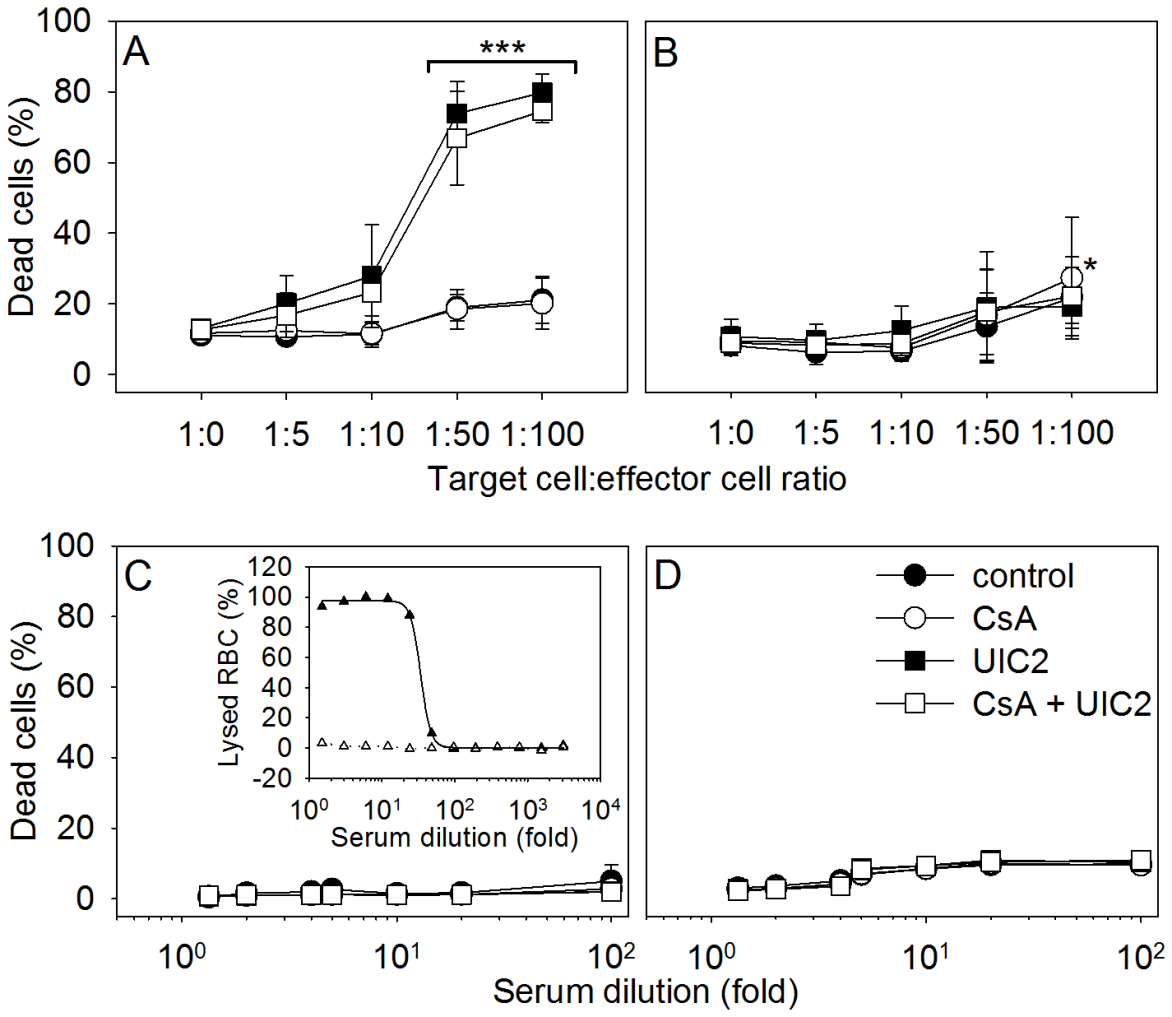
Antibody-dependent cell-mediated cytotoxicity (ADCC, *panels A* and *B*) and complement mediated lysis (CDC, *panels C* and *D*) induced by UIC2 mAb treatment *in vitro*. In the ADCC assay KB-V1 (*A*) and KB-3-1 (*B*) tumor cells were labeled with CFDA-SE then mixed with PBMCs freshly isolated from peripheral blood at different target to effector cell ratios. Samples were treated with 10 µM CsA (○), 20 µg/ml UIC2 mAb (▪), 10 µM CsA and 20 µg/ml UIC2 mAb (□) or buffer (•). After 8 h incubation at 37°C, samples were stained with PI and analyzed by flow cytometry. In the CDC assay, cells were incubated with human serum at different dilutions for 4 h. *Inset of panel C*: Hemolytic effect of the serum (▴) and of heat inactivated serum (▵) on sensitized sheep red blood cells served as positive and negative control, respectively. The percentages of killed cells were calculated as described in Materials and Methods. Values are means (± SD) of four independent experiments, ***P<0,001.

Fig. 6 shows the effect of human peripheral blood mononuclear cells (PBMCs), and of human serum samples, on UIC2 mAb treated KB-V1 and KB-3-1 cells, *in vitro*. PBMCs killed about 70-80% of the UIC2 treated KB-V1 cells both in the presence and absence of CsA, at target to effector cell ratios of 1∶50 and 1∶100, respectively (Fig. 6*A*), in contrast with the UIC2 treated KB-3-1 cells that exhibited a survival rate similar to that of the untreated control (Fig. 6*B*). In the absence of UIC2, the percentages of dead target cells were low (see Fig. 6*A and B*).

In order to assess the possible role of CDC in UIC2 mediated *in vivo* tumor cell killing, the cytotoxicity of human serum samples was measured *in vitro*. Cell killing did not increase in the UIC2 treated KB-V1 (Fig. 6*C*) and KB-3-1 (Fig. 6*D*) samples despite the strong hemolytic activity of the applied sera (Fig. 6*D* *, inset*).

## Discussion

In the present experiments SCID mice xenotransplanted with Pgp^+^ and Pgp^-^ tumors were used to study the efficacy of an antibody-based multidrug resistance reversal strategy. The KB-V1/KB-3-1 cell pair does not express ABCG2 and MRP1 (ABCC1) at detectable levels, while the KB-V1 cells have high Pgp expression level (see Fig. S2 and Fig. 2). They are growing fast and develop into subcutaneous tumors of ∼1 cm diameter in about 10-12 days after inoculation of 1–3×10^6^ cells into the animals. An advantage of the fast tumor growth in this model system is that the Pgp expression level of the tumor cells does not decline in the absence of Pgp substrates (see Fig. 2) on the time scale of the *in vivo* experiments.

In the above model system, co-treatment with UIC2 + CsA potentiated the anti-tumor effect of DOX and inhibited or hindered the development of KB-V1 Pgp^+^ tumors *in vivo* (Fig. 4). At the same time, DOX treatment alone did not have a significant effect on the size of the KB-V1 tumors. These data are in line with the conclusions of our previously published *in vitro* and *in vivo* drug accumulation studies [22] and with the results of the *in vitro* cytotoxicity measurements shown in Fig. 1. Although these observations may suggest that the dramatic antitumor effect of the combined treatment is the result of increased antibody binding with consequential Pgp inhibition and DOX accumulation, the mechanism proved more complex.

SCID mice have intact complement system as well as functioning macrophages, natural killer cells and polymorphonuclear cells [32]. Therefore, antibody binding to the tumor cells may elicit cytotoxicity directly through complement binding (complement-dependent cytotoxicity, CDC) or indirectly, via the recruitment of the above effector cells to the antibody covered tumor cells (antibody-dependent cell-mediated cytotoxicity, ADCC). The possibility, that immunological factors contribute to the cytotoxic effect of our treatment protocol is strongly supported by the difference between the outcomes of *in vitro* cytotoxicity measurements and of the *in vivo* experiments conducted in SCID mice (compare Fig.1 and Fig. 4). In the *in vitro* cytotoxicity assay, UIC2 alone only mildly aggravated DOX cytotoxicity (see Fig. 1) what could be explained by the trapping and inhibition of only a small fraction of cell surface Pgps by UIC2 in the absence of CsA [22]. In contrast, we experienced *in vivo* a marked decrease of tumor size in response to the DOX+UIC2-only treatment, as the average weight of the KB-V1 tumors was approx. 4 fold smaller compared to the animals treated with DOX alone (Fig. 4*A*). These data are in line with the positive results of our *in vitro* ADCC assays (Fig. 6), supporting the notion that the UIC2 mAb also induces ADCC *in vivo* in the SCID mice.

The involvement of ADCC in the *in vivo* anti-tumor effect of UIC2 treatment is an unexpected finding of our experiments, since IgG_2_ antibodies are mostly inefficient at supporting effector functions and are chosen for antibody therapy when effector functions are unnecessary or undesirable [33]. However, there is an example [34] when the antitumor and antimetastatic effects of two IgG_2_ isotye anti-Pgp antibodies (the mouse-human chimeric Ab (MH162) and its mouse counterpart (MRK16)) was attributed to ADCC. Similarly, it was proven in a recent study that a human IgG_2_ isotype mAb specific for epidermal growth factor receptor effectively triggers ADCC by recruting monocytes and neutrophils [35] via FcγRIIa binding [36], [37]. Since IgG_2_ isotype antibodies do not trigger natural killer cell mediated ADCC [35], therefore in our *in vitro* ADCC experiments carried out with PBMCs cell killing was mediated by monocytes. Since SCID mice also have monocytes [32] the same mechanism is functional and probably explains our *in vivo* results.

The strong anti-tumor effect of the combined treatment might be attributed exclusively to ADCC triggered by the UIC2 mAb binding. However, the fact that the extents of the *in vitro* ADCC effects were indistinguishable in the presence of UIC2 or UIC2+CsA suggests that binding of the antibody to a small fraction of the cell surface Pgps (20-40%) is sufficient to induce a maximal ADCC effect (Fig. 6*A*). Consequently, the differences in the size of the KB-V1 tumors between the UIC2 and UIC2+CsA treated animals and the lack of the KB-V1 tumors in 52% of these animals (Fig 4*A and B*) argue against the above assumption and suggests that the stronger Pgp inhibitory effect of the UIC2+CsA combination mediates the anti-tumor effect at least in part. Pgp inhibition by the antibody requires saturating antibody concentrations that seems to be reached in our experiments, since strong UIC2 staining and a two fold increase in the accumulation of a Pgp substrate daunorubicin was measured in the tumor sections prepared from the tumors 8 hours after the injection of UIC2 and CsA [22] added at similar conditions. Taken together, our *in vitro* and *in vivo* data suggest that strong anti-tumor effect can be reached by the combinative treatment studied, as a joint result of Pgp inhibition and ADCC. However, the relative contribution of these mechanisms to the anti-tumor effect of the UIC2 mAb is not known.

ADCC can be triggered at relatively low receptor occupancy by the antibody or at low receptor abundance [35], [38]. Thus, the 60 fold increased Pgp expression level of the KB-3-1 tumors compared to the KB-3-1 cells (see Fig. 2.) is probably sufficient to trigger ADCC effect, when the Pgp molecules are saturated by the antibody in the presence of CsA. In line with this assumption in 52% of the DOX-UIC2-CsA treated animals we could not detect KB-3-1 tumors, while they appeared in all of the DOX or DOX+UIC2 treated animals (see Fig. 4*B*).

It could be a limitation of our study that only one cell line pair was used in the experiments. However, the observed effects have been proven to be Pgp specific, since decreased tumor size was detected exclusively in those animal groups that received UIC2 mAb treatment. In addition, in our previous studies we compared the KB-V1 cell line with mdr1 transfected NIH 3T3 murine fibroblast cells [22], and Pgp^+^ A2780^AD^ ovarian carcinoma cells [23] and found them equivalent in every aspect of their multidrug resistant phenotype including the inhibition of Pgp-mediated drug transport by UIC2 mAb. On the other hand, ADCC effect is not dependent on the tissue origin of the target cells; rather it is determined by the interaction between the Fc part of the antibody and the Fc receptor of the effector cells.

Doubts about the possible clinical application of an anti-Pgp mAb based tumor therapy are related to the likely side effects that may arise as a result of either Pgp inhibition or ADCC, both exerted on cells expressing Pgp at physiological barriers of the body. For instance, inhibition of Pgp expressed in the blood-brain barrier may lead to increased accumulation of its substrates in the central nervous system leading to neurotoxicity, as it was experienced in mdr1a/b knock-out mice [8]. However, administration of Pgp modulators in clinical trials does not seem to cause toxicity to the central nervous system [39] probably because other ABC transporters (e.g. MRP1, BCRP1) may compensate for the loss off Pgp's gatekeeper function in the blood-brain barrier [40]. However, in contrast to Pgp inhibition, ADCC may damage the tissues at the physiological Pgp expression sites. Our *in vitro* assays using human PBMCs confirmed that UIC2 mAb effectively triggers ADCC. Since ADCC is mediated via the Fc portion of the antibody, to avoid this side-effect upon human applications the whole UIC2 antibody could be substituted for by its Fab fragments (that behave very similarly to whole antibodies *in vitro*; our unpublished data) or upon humanization of the antibody its effector functions may be fine-tuned by the design of the Fc part [33]. However, it remains to be studied if inhibition of Pgp function alone may augment DOX cytotoxicity sufficiently enough for a therapeutically significant anti-tumor effect.

The UIC2 mAb does not bind to mouse Pgp [14], therefore the SCID mouse model system is not applicable for studying the possible side effects brought about by antibody binding to physiological Pgp expression sites. Since the UIC2 mAb also recognizes primate [14] and sheep [41] Pgp, such animal models may be used for the evaluation of the feasibility of the strategy demonstrated herein. Direct injection of the antibody into the tumor tissue may also be tested for the purposes of reducing antibody dose and decrease systemic side effects.

In our model system, treatments were applied shortly (four days) after injection of the tumor cells, when the tumors were still rather small, a situation perhaps analogous to the clinical setting when systemic therapy is applied to prevent or hinder the development of multidrug resistant primary or metastatic tumors.

Each multidrug resistant tumor may have a unique signature of resistance mechanisms. Consequently, cancer therapy will need to be personalized, not only with respect to the mechanisms of malignant transformation but also regarding the mechanisms of resistance [42]. The strategy of Pgp inhibition demonstrated herein is offered to enrich the repertoir of possible protocols that can be considered for the treatment of multidrug resistant tumors, once humanized UIC2 becomes available.

## Supporting Information

Figure S1 **In the ADCC assay KB-V1 (panels** ***A*** **,** ***B*** **,** ***C*** **) and KB-3-1 (** ***D*** **,** ***E*** **,** ***F*** **) tumor cells were labeled with CFDA-SE, then mixed with PBMCs freshly isolated from peripheral blood at 1∶5 (** ***B*** **,** ***E*** **) or 1∶100 (** ***C*** **,** ***F*** **) target to effector cell ratios.** Samples were treated with 20 µg/ml UIC2 mAb. After 8 h incubation at 37°C, samples were stained with PI and analyzed by flow cytometry. *Green gates* mark living target cells, while *red gates* show dead target cells.
(TIF)

Figure S2 **Flow cytometric analysis of Pgp, ABCG2 and MRP1 expression in KB-V1 (** ***A*** **,** ***C*** **,** ***E*** **) and KB-3-1 (** ***B*** **,** ***D*** **,** ***F*** **) cell lines applying indirect immunofluorescence staining by CFL647-conjugated GaMIgG.** Pgp was detected by 15D3 mAb, while ABCG2 and MRP1 (ABCC1) were labeled by 5D3 and QCRL-3 mAbs, respectively. Grey filled histograms show antibody untreated cells, while dashed lines represent samples treated with secondary antibody only. The ABCG2 positive MDCK ABCG2 cells (*G*) and the MRP1 (*I*) expressing GLC4-ADR cell line and their nonexpressing counterparts (*H* and *J*) were used as controls. The GLC4 human small cell lung carcinoma cell line pair was a kind gift from Pinedo HM (Department of Medical Oncology, Academic Hospital Vrije Universiteit, Amsterdam), while the MDCK (Madin-Darby canine kidney) cell line and its ABCG2 transfected counterpart was kindly provided by Sarkadi B (Biomembrane Institute of Molecular Pharmacology, Budapest)
(TIF)

Figure S3 **Pgp expression of KB-V1 (** ***A*** **) and KB-3-1 (** ***C*** **) tumor xenografts visualized by indirect immunofluorescence.** Panels (*B*) and (*D*) are phase contrast images of the same tumor slices. The 5 µm thick cryosections were fixed in acetone, labeled with UIC2 mAb followed by A488-GaMIgG at room temperature for 60 min.
(TIF)
