# Supplementary figures and images for: The Strong In Vivo Anti-Tumor Effect of the UIC2 Monoclonal Antibody Is the Combined Result of Pgp Inhibition and Antibody Dependent Cell-Mediated Cytotoxicity
Source: PLoS One. 2014 Sep 19;9(9):e107875. doi: 10.1371/journal.pone.0107875 (PMC4169599; doi:10.1371/journal.pone.0107875)

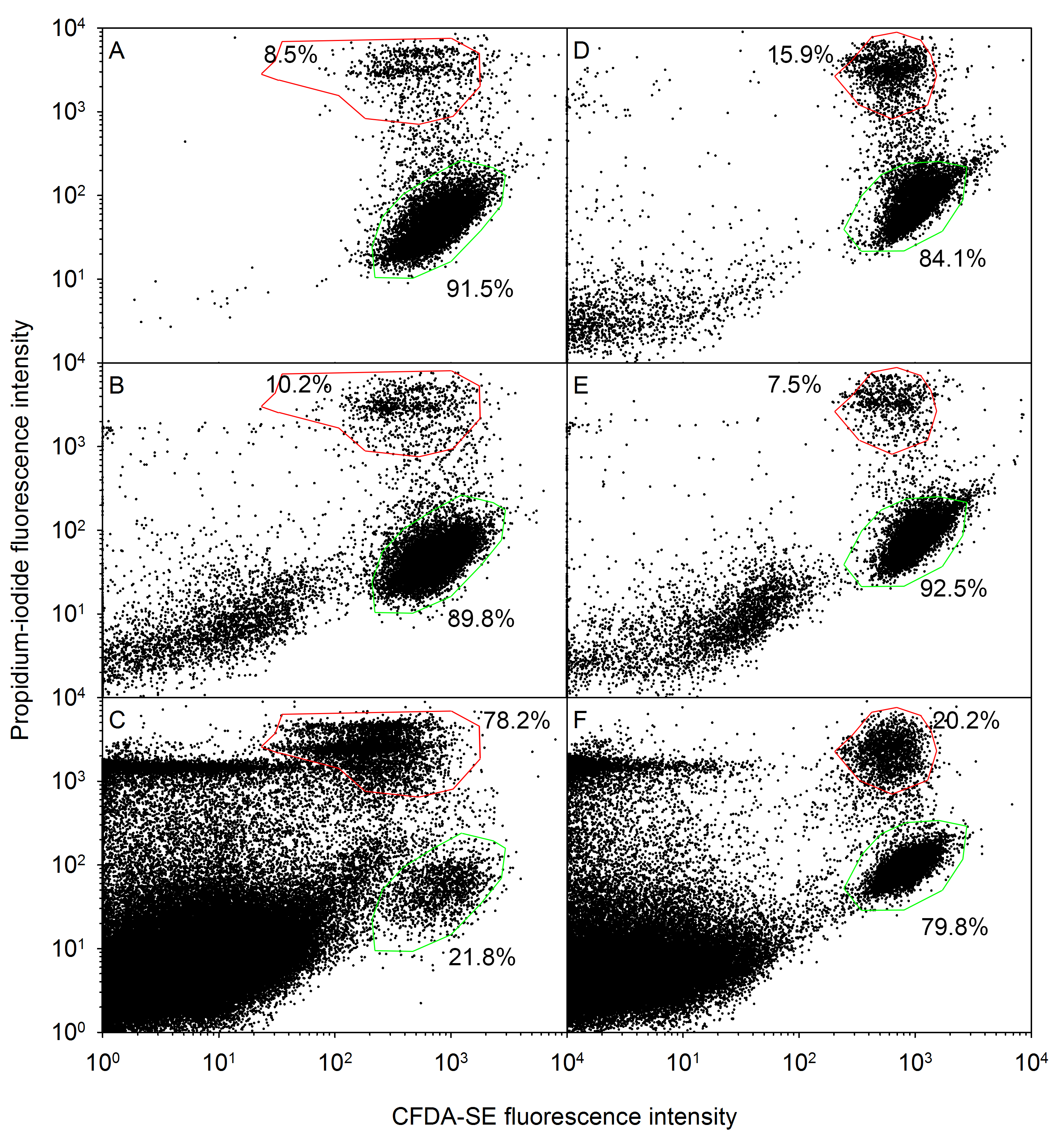

Supplement: Figure S1 — In the ADCC assay KB-V1 (panels A , B , C ) and KB-3-1 ( D , E , F ) tumor cells were labeled with CFDA-SE, then mixed with PBMCs freshly isolated from peripheral blood at 1∶5 ( B , E ) or 1∶100 ( C , F ) target to effector cell ratios. Samples were treated with 20 µg/ml UIC2 mAb. After 8 h incubation at 37°C, samples were stained with PI and analyzed by flow cytometry. Green gates mark living target cells, while red gates show dead target cells. (TIF) [file pone.0107875.s001.tif]

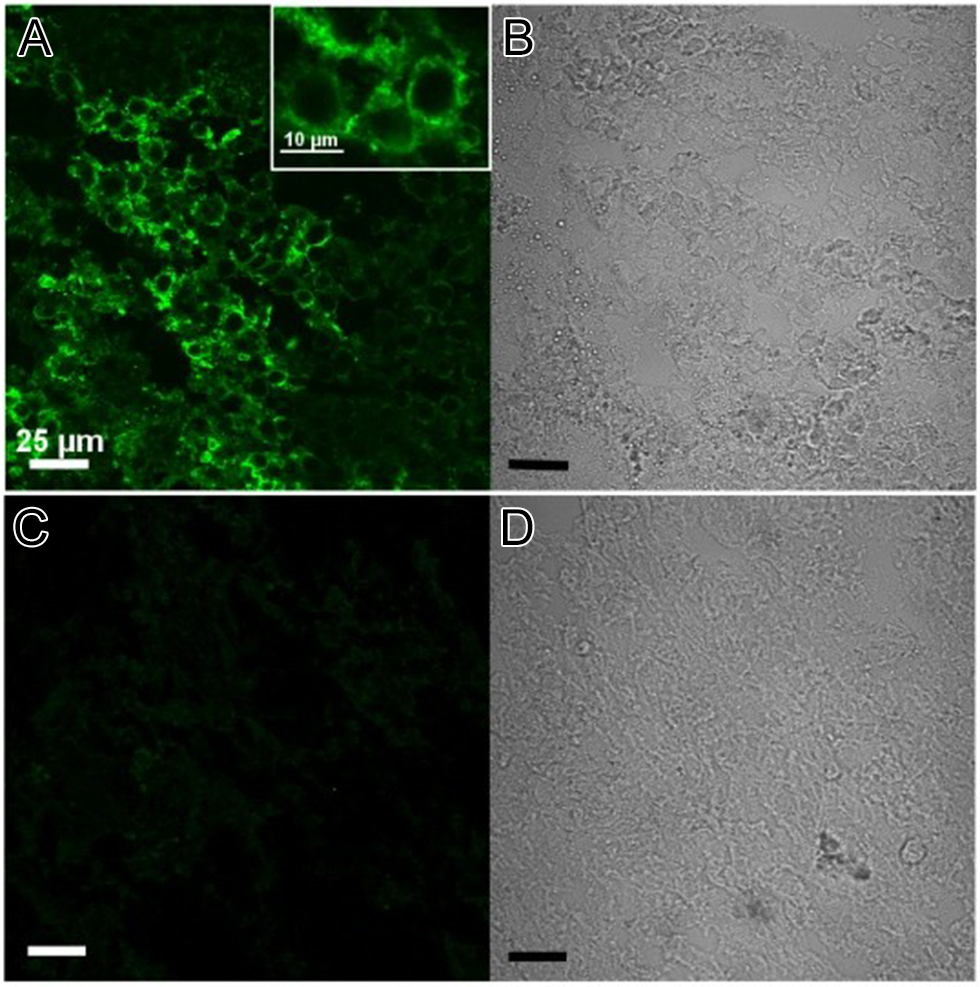

Supplement: Figure S3 — Pgp expression of KB-V1 ( A ) and KB-3-1 ( C ) tumor xenografts visualized by indirect immunofluorescence. Panels (B) and (D) are phase contrast images of the same tumor slices. The 5 µm thick cryosections were fixed in acetone, labeled with UIC2 mAb followed by A488-GaMIgG at room temperature for 60 min. (TIF) [file pone.0107875.s003.tif]
